# IgG Antibody Responses to the *Aedes albopictus* 34k2 Salivary Protein as Novel Candidate Marker of Human Exposure to the Tiger Mosquito

**DOI:** 10.3389/fcimb.2020.00377

**Published:** 2020-07-29

**Authors:** Sara Buezo Montero, Paolo Gabrieli, Fabrizio Montarsi, Alessio Borean, Stefano Capelli, Giustina De Silvestro, Federico Forneris, Marco Pombi, Antonio Breda, Gioia Capelli, Bruno Arcà

**Affiliations:** ^1^Division of Parasitology, Department of Public Health and Infectious Diseases, Sapienza University of Rome, Rome, Italy; ^2^Department of Biology and Biotechnology “L. Spallanzani”, University of Pavia, Pavia, Italy; ^3^Laboratory of Parasitology, Istituto Zooprofilattico Sperimentale delle Venezie, Legnaro, Italy; ^4^Department of Immunohematology and Transfusion Medicine, San Martino Hospital, Belluno, Italy; ^5^Department of Transfusion Medicine, Padua University Hospital, Padova, Italy; ^6^Coordinamento Regionale Attività Trasfusionali (CRAT), Padova, Italy

**Keywords:** *Aedes albopictus*, 34k2 salivary protein, human exposure, marker, vector control, arboviruses, epidemiological tool

## Abstract

Mosquitoes of the *Aedes* genus transmit arboviruses of great importance to human health as dengue, chikungunya, Zika and yellow fever. The tiger mosquito *Aedes albopictus* can play an important role as arboviral vector, especially when *Aedes aegypti* is absent or present at low levels. Remarkably, the rapid worldwide spreading of the tiger mosquito is expanding the risk of arboviral transmission also to temperate areas, and the autochthonous cases of chikungunya, dengue and Zika in Europe emphasize the need for improved monitoring and control. Proteomic and transcriptomic studies on blood feeding arthropod salivary proteins paved the way toward the exploitation of genus-specific mosquito salivary proteins for the development of novel tools to evaluate human exposure to mosquito bites. We previously found that the culicine-specific 34k2 salivary protein from *Ae. albopictus* (al34k2) evokes specific IgG responses in experimentally exposed mice, and provided preliminary evidence of its immunogenicity to humans. In this study we measured IgG responses to al34k2 and to *Ae. albopictus* salivary gland protein extracts (SGE) in individuals naturally exposed to the tiger mosquito. Sera were collected in two areas of Northeast Italy (Padova and Belluno) during two different time periods: at the end of the low- and shortly after the high-density mosquito seasons. Anti-SGE and anti-al34k2 IgG levels increased after the summer period of exposure to mosquito bites and were higher in Padova as compared to Belluno. An age-dependent decrease of anti-saliva IgG responses was found especially in Padova, an area with at least 25 years history of *Ae. albopictus* colonization. Moreover, a weak correlation between anti-saliva IgG levels and individual perception of mosquito bites by study participants was found. Finally, determination of anti-al34k2 IgG1 and IgG4 levels indicated a large predominance of IgG1 antibodies. Overall, this study provides a convincing indication that antibody responses to al34k2 may be regarded as a reliable candidate marker to detect temporal and/or spatial variation of human exposure to *Ae. albopictus*; a serological tool of this kind may prove useful both for epidemiological studies and to estimate the effectiveness of anti-vectorial measures.

## Introduction

Arboviruses as dengue, chikungunya, Zika and yellow fever have been responsible for severe outbreaks in the last decades, causing tens of thousands deaths per year with heavy public health impact and important global economic losses (Wilder-Smith et al., 2017). These four major arboviruses are transmitted by mosquitoes of the *Aedes* genus, with the most important competent vectors being by far the yellow fever mosquito *Aedes aegypti* and the tiger mosquito *Aedes albopictus*. Due to globalization and environmental changes, their distribution is rapidly and progressively expanding into new tropical, subtropical and temperate areas (Kraemer et al., 2015, 2019). Even though *Ae. aegypti* is the main vector of these arboviruses, *Ae. albopictus* can play an important role as an epidemic driver, especially in areas where *Ae. aegypti* is absent or present at low levels. This has been the case for the large chikungunya (2005) and dengue (2018) outbreaks in the Reunion Island (Renault et al., 2007; Vincent et al., 2019), for the several cases of dengue and chikungunya autochthonous transmission (2007–2018) in Italy, France and Croatia (Gossner et al., 2018), or for the more recent cases of Zika virus transmission in southern France (Brady and Hay, 2019; Giron et al., 2019). Importantly, there are currently no specific anti-viral drugs to treat these diseases. A dengue vaccine has been approved by FDA in 2019 but its use appears to have some limitations (CDC, 2019; Espana et al., 2019), and despite the availability of a safe and effective vaccine for yellow fever, the disease is still endemic in Africa and in Central-South America (WHO, 2019). In this scenario, vector monitoring and control, along with the prevention of human-mosquito contact, still represent the main methods to contain the transmission of these arboviral diseases.

Evaluation of human-vector contact is essential to assess the risk of transmission of mosquito-borne diseases and to guide planning and implementation of vector control by public health authorities. For *Aedes* mosquitoes this is currently obtained by entomological methods as ovitraps, larval/pupal indices, adult traps or human landing catches (HLC), which provide estimates of adult and/or immature mosquito densities in a given area (ECDC, 2012). However, entomological measurements have some limitations and drawbacks. First, they only provide an indirect estimation of human exposure to vectors at community level. Second, they can be expensive, labor-intensive and/or difficult to carry out in some epidemiological settings (e.g., logistic constraints or low vector densities) or may raise ethical issues (e.g., for HLC). In addition to classical entomological methodologies, a novel tool for the evaluation of human exposure to disease vectors is emerging. This alternative approach, which allows for a direct estimation of human-vector contact at the individual level, relies on the assessment of host antibody responses against mosquito salivary proteins injected by hematophagous arthropods during blood feeding (Ribeiro and Arcà, 2009). As first shown for ticks (Schwartz et al., 1990), and then for several other blood feeders including anopheline and culicine mosquitoes (Trevejo and Reeves, 2005; Remoue et al., 2006; Doucoure et al., 2012b), these antibody responses can be used to evaluate human exposure to arthropod vectors. Transcriptomic and proteomic studies performed in the last two decades allowed to unravel the complexity of the salivary repertoires of blood feeding insects (Arcà and Ribeiro, 2018) and to identify groups of genus-specific mosquito salivary proteins, i.e., only found in the saliva of either anopheline or culicine mosquitoes (Ribeiro et al., 2010). These findings provided the starting point for shifting from the use of saliva or salivary gland protein extracts (SGE) to the exploitation of individual genus-specific mosquito salivary proteins. In fact, the use of saliva/SGE is inconvenient (difficult to obtain in large amounts) and may even be misleading (content variation according to physiological states, possible cross-reactions). On the other side, genus-specific salivary proteins may represent ideal candidates for the development of immuno-assays suitable for the evaluation of human exposure to either *Anopheles* or *Aedes* vectors.

A solid proof of principle has been already provided for anopheline malaria vectors. IgG responses to the *Anopheles gambiae* gSG6 or the gSG6-P1 peptide have been widely validated as marker of human exposure to Afrotropical malaria vectors in different epidemiological settings in Western and Eastern African countries (Poinsignon et al., 2008; Drame et al., 2010; Rizzo et al., 2011b; Badu et al., 2012; Stone et al., 2012; Proietti et al., 2013; Yman et al., 2016). Moreover, the same antigens have been successfully employed for Asian malaria vectors (Ya-Umphan et al., 2017) and may be of some use with Polynesian (Idris et al., 2017; Pollard et al., 2019) and American Old World anophelines (Montiel et al., 2020). For *Aedes* mosquitoes some promising indications have been obtained with the Nterm-34kDa peptide, which is designed on the culicine-specific *Ae. aegypti* 34k1 salivary protein (Sagna et al., 2018). This peptide allowed to detect variation of human exposure to *Ae. aegypti* bites in studies in Benin, Côte d'Ivoire and Lao PDR (Elanga Ndille et al., 2012; Ndille et al., 2014; Yobo et al., 2018). Moreover, it proved useful to evaluate vector control intervention at La Reunion Island (Indian Ocean), in an urban area where *Ae. aegypti* is not present and individuals are only exposed to *Ae. albopictus* (Elanga Ndille et al., 2016), suggesting it may be of use to assess exposure to both these vectors.

Toward the development of novel additional markers of human exposure to *Aedes* mosquitoes, and more specifically to *Ae. albopictus*, we previously analyzed in a murine model the immunogenic properties of the *Ae. albopictus* 34k2 salivary protein (al34k2). It is worth pointing out that this protein, as the 34k1 mentioned above, is also culicine-specific and a member of the 34kDa salivary protein family. Nevertheless, 34k1 and 34k2 proteins are significantly divergent and only share 32 and 33% amino acid identity in *Ae. albopictus* and *Ae. aegypti*, respectively (Arcà et al., 2007; Ribeiro et al., 2007). We found specific anti-al34k2 IgG responses in mice experimentally exposed to bites of *Ae. albopictus* and in a single human donor hyperimmune to *Ae. albopictus* saliva (Buezo Montero et al., 2019). Moreover, mice immunized by exposure to bites of *Ae. aegypti* had no IgG antibodies cross-reacting to al34k2, suggesting it may be useful for the development of species-specific immunoassays. To follow up these initial observations we analyzed in this study a set of sera collected from healthy human blood donors in two areas with different levels of natural exposure to *Ae. albopictus* (Padova and Belluno, Northeast Italy) during two different periods, at the end of the low-density mosquito season and shortly after the high-density mosquito season.

## Materials and Methods

### Ethical Statement

This study was conceived according to good clinical practices and followed the ethical principles recommended by the Edinburgh revision of the Helsinki declaration. The protocol was approved by the Ethics Committee of Sapienza University (306/17 RIF.CE: 4479, April 10th 2017). All volunteers participating to the study provided written informed consent on the use of their sera to measure antibody responses to mosquito salivary antigens.

### Study Areas and Sera Collections

The study was carried out in the Veneto region, Northeast Italy, in the cities of Padova and Belluno (Supplementary Figure 1A). Padova (45°24′23”N, 11°52'40”E) is located in a plain area (27 meters a.s.l.), has a relatively high population density (2.287 inhabitants/km^2^) and counts roughly 213,000 inhabitants. Belluno (46°08′27″N, 12°12′56″E) is situated in a valley at 389 meters a.s.l. and is surrounded by Bellunesi Prealps and Dolomites; total population is of approximately 36,000 inhabitants with a population density lower than Padova (243 inhabitants/km^2^). *Aedes albopictus* is widely spread almost all over Northeast Italy and Padova is one of the first cities in Europe colonized by this species. After its first finding in 1991 (Dalla Pozza and Majori, 1992), the tiger mosquito got very well established in the area and quickly became an important pest due to its aggressive behavior and daytime biting activity. Afterwards, the tiger mosquito progressively expanded its distribution to the entire Veneto region. Currently, it is by far the most abundant *Aedes* species in the urban areas of Italy, and these two cities were selected as sites with high (Padova) and low to moderate (Belluno) exposure to bites of *Ae. albopictus*. This assumption was mainly based on entomological data from the two areas in the years preceding this study (Montarsi et al., 2015; Montarsi F., unpublished observations) and on history of colonization. In fact, even though *Ae. albopictus* is well established in both municipalities, the two sites markedly differ for the timing of colonization. Padova should be considered of “ancient” colonization: the tiger mosquito was first reported at the beginning of nineties (Dalla Pozza and Majori, 1992) and therefore, at the time of this study, it was established in the city since at least 25 years. On the contrary, Belluno is of “recent” colonization: *Ae. albopictus* reached the area approximately 20 years later, in 2012 (Gobbi et al., 2014), and for this reason at the time of our study was established in Belluno since approximately 5 years. Notably, another exotic mosquito species, *Aedes koreicus*, was found shortly earlier in the Belluno area (Capelli et al., 2011); however, according to entomological surveys performed in the period 2014–2015, the most abundant mosquito species in the Belluno city was *Ae. albopictus* (57%), followed by *Culex pipiens* (32.1%) and *Ae. koreicus* (9.2%), with other *Aedes* species only occasionally found and accounting globally for less than 0.8% of the collected mosquitoes (Baldacchino et al., 2017). It should be also mentioned that another mosquito of Asian origin, *Aedes japonicus japonicus*, was found in 2018 in the far Northeast area of the Belluno province, toward the borders with Austria (Montarsi et al., 2019); however, there is no indication of the presence of *Ae. j. japonicus* in Belluno during the study period.

Planning the sample size for sera collection was not straightforward considering the absence of information on the anti-saliva IgG response in the study sites and the novelty of the al34k2 antigen. A rough estimation was done by conservatively considering an unpaired sample and taking into account previous similar studies (Elanga Ndille et al., 2012, 2016). Hypothesizing for all the comparisons a similar difference of average IgG levels not lower than 0.15 and a variance of 0.16, a minimum number of 112 sera per group could guarantee a power of 80% with a 95% confidence level. In order to increase the power up to approximately 85% we collected around 130 samples/group. Sera were collected among adult healthy volunteers who referred for routine blood donation to the immune transfusion centers of Padova (Padova University Hospital) and Belluno (San Martino Hospital), both located in the Veneto region. A first collection of sera took place in 2017 from May 2nd to May 12th in Padova (PD1, *n* = 130) and from May 4th to June 1st in Belluno (BL1, *n* = 130). According to previous data on mosquito seasonality in the areas (Baldacchino et al., 2017), these sera can be considered as representative of individuals who were not significantly exposed to *Ae. albopictus* bites in the previous 4–5 months. We will refer to these collections in the text as PD1 and BL1 or, more generically, as before (summer) = at the end of the low-density mosquito period. A second collection was done, always in 2017, after the summer period of high mosquito density: from September 11th to November 22nd in Padova (PD2, *n* = 132) and from September 14th to November 21st in Belluno (BL2, *n* = 131). These sera can be considered as representative of individuals who were significantly exposed to *Ae. albopictus* bites. In the text we will refer to this second round of collections as PD2 and BL2 or, more generically, as after (summer) = after the high-density mosquito period. A subset of individuals from Padova (*n* = 69) and Belluno (*n* = 97) could be enrolled in both surveys. Volunteers participating to the study were also invited to fill a short questionnaire finalized to gather information on (i) cutaneous reaction to mosquito bites (from 0 = absent to 5 = very intense) as well as, with specific reference to the 6-months preceding the donation, on (ii) travels outside Italy and country visited, (iii) perception of intensity of mosquito bites (from 0 = not bitten to 5 = very many bites) and (iv) timing of mosquito bites (during day, at night, day and night).

### Entomological Measurements

To evaluate the occurrence and population density of *Ae. albopictus* the two selected sites, Padova and Belluno, were monitored from end of May to July 2017 (low-density) and from end of August to beginning of October 2017 (high-density). The surveys were performed using ovitraps (oviposition standard traps), which is the most used kind of trap for monitoring *Aedes* mosquito species (Velo et al., 2016; Manica et al., 2017). Ovitraps consist of black cylindrical vessels (9.0 × 11.0 cm) with an overflow hole (at 7.0 cm from the bottom) containing ~300 mL of standing water. A wooden stick (Masonite strip, 10.0 × 2.5 cm) was used as a substrate for oviposition. A larvicide (*Bacillus turingensis* var. *israelensis*, BTI) was added into the ovitraps to avoid larval development. Selection of sites where to set the ovitraps was made by dividing the urban areas into hypothetical squares of 4 km^2^ and positioning three traps inside each square. According to these criteria, ovitraps were placed in geo-referenced sites and checked biweekly, with a total of 20 ovitraps in Belluno and 40 in Padova (Supplementary Figures 1B,C). The mean number of eggs per positive ovitraps and the proportion of positive ovitraps (number of traps with eggs over total number of ovitraps) were calculated to estimate the seasonal mosquito density.

### Mosquito Rearing, Salivary Gland Extracts and 34k2 Salivary Protein

*Aedes albopictus* mosquitoes (originally collected in Rome, Italy) were reared in the insectary of Sapienza University at 27 ± 1°C, 70 ± 10% relative humidity, 14:10 h light:dark photoperiod. Adult females 3–8 days post-emergence (dpe), and never fed on blood before, were used for all the experiments. Salivary glands were dissected in Phosphate Buffered Saline (PBS), transferred to a microcentrifuge tube kept on ice and containing 20 μl of PBS, then frozen at −80°C (typically in batches of 20–40 salivary gland pairs). *Aedes albopictus* salivary gland protein extracts (SGE) were prepared by three cycles of freezing and thawing, followed by centrifugation at 16,000 × g at 4°C. Supernatants were pooled in order to generate a homogeneous SGE stock to be used for all ELISA assays; protein concentration was measured by the Bradford method (Bio-Rad, 5000002) using the Take3 micro-volume plate in a BioTek microplate reader (BioTek Synergy HT). SGE stocks were stored at −20°C in small aliquots until use. The *Ae. albopictus* al34k2 salivary protein was expressed and purified as previously described (Buezo Montero et al., 2019).

### Serological Tests

Enzyme-linked immunosorbent assays (ELISA) were essentially performed as previously described (Rizzo et al., 2011a, 2014a; Buezo Montero et al., 2019). Briefly, flat-bottom 96-well plates (Nunc MaxiSorp, 442404) were coated for 3 h at room temperature (RT) with 50 μl of either *Ae. albopictus* SGE (5.6 μg/ml, equivalent to the amount of proteins per ml obtained from 7 salivary gland pairs) or recombinant al34k2 salivary protein (5 μg/ml) diluted in Coating Buffer (15mM Na_2_CO_3_, 35mM NaHCO_3_, 3mM NaN_3_, pH 9.6). Afterwards plates were: (i) blocked for 3 h at RT (150 μl 1% w/v skimmed dry milk in PBST, i.e., PBS plus 0.05% Tween 20); (ii) incubated overnight at 4°C with 50 μl of plasma diluted 1:50 (IgG) or 1:20 (IgG1 and IgG4); (iii) incubated for 3 h at RT with 100 μl of polyclonal rabbit anti-human IgG/HRP (Dako P0214, dilution 1:5000) or sheep anti-human IgG1/HRP (Binding Site AP006, dilution 1:1000) or sheep anti-human IgG4/HRP (Binding Site AP009, dilution 1:1000); (iv) incubated in the dark at 25°C for 15 min with 100 μl of o-phenylenediamine dihydrochloride (OPD, Sigma P8287) for colorimetric development. Reactions were terminated by adding 25 μl of 2M H_2_SO_4_. Three to four washings were performed between each step. OD_492_ were determined using a microplate reader (Biotek Synergy HT).

### Data Normalization and Analysis

All samples were analyzed in duplicate with the antigen and once with no antigen. The no-antigen well was used for background subtraction, and IgG levels were expressed as final OD calculated for each sample as the mean OD value with antigen minus the OD value without antigen. Samples whose duplicates showed a coefficient of variation (CV) >20% were re-assayed or not included in the analysis. To control for intra- and inter-assay variation, IgG levels were determined including in each plate negative controls as well as a standard curve made by 2-fold dilution series (1:25–1:1,600) of a human hyperimmune serum. OD values were normalized using titration curves and the Excel software (Microsoft) with a three variable sigmoid model and the Solver add-in application as previously described (Corran et al., 2008). IgG1 and IgG4 OD levels were converted to concentrations (ng/ml) including on each plate standard curves set up as follows. As capturing factors goat anti-human IgG (5 μg/ml, Jackson ImmunoResearch 109005098) or mouse anti-human IgG4 (2 μg/ml, BD Pharmingen 555881) were used for coating (in 50 μl coating buffer, 3 h at RT). After washing, blocking and washing again, wells were incubated overnight at 4°C with two-fold dilution series from 1 μg/ml to 0.0078 μg/ml of purified native human IgG1 (Bio-Rad PHP010) or IgG4 (ABD Serotec 5254–3004) in 50 μl of blocking reagent. Incubation with anti-human IgG1/HRP or IgG4/HRP and colorimetric detection were performed as described above. All datasets were tested for normality and lognormality by different tests (Anderson-Darling, D'Agostino & Pearson, Shapiro-Wilk, Kolmogorov-Smirnov). No dataset passed any normality test and only some datasets passed lognormality tests. For these reasons the statistical analysis was performed using non-parametric tests. Multiple comparisons were performed by the Kruskal-Wallis test. Mann-Whitney *U*-test was used to compare IgG levels between two independent groups. The Wilcoxon matched-pairs test was used for comparison of two paired groups. Graph preparation and statistical analyses were performed using the Prism 8.0 GraphPad Software (San Diego, CA).

## Results

The main characteristics of the studied population and the individual perception of mosquito bites are summarized in Table 1. Collected sera were used to measure IgG responses to *Ae. albopictus* salivary gland protein extracts (SGE) and to the recombinant *Ae. albopictus* salivary protein al34k2 (Buezo Montero et al., 2019). Considering that male volunteers were largely predominant, and to make sure not to introduce any bias, we preliminarily compared IgG responses to SGE in males versus females and found no statistically significant difference (Supplementary Figure 2A); similar results were obtained comparing anti-al34k2 IgG responses in the two sexes. Moreover, in the 6-months preceding the surveys a variable proportion of individuals (16.1–28.8%) had traveled to countries where *Ae. albopictus* was either present or absent. No significant variation of anti-SGE IgG levels was found by pairwise comparisons between individuals who did not travel and those who: (i) traveled, (ii) traveled to countries where *Ae. albopictus* was present or (iii) traveled to countries where *Ae. albopictus* was absent (Supplementary Figure 2B). According to these observations, the analyses described below were performed including all the samples collected in the different surveys.

**Table 1:** Features of the studied population and individual perception of mosquito bites.

		**PD1**	**PD2**	**BL1**	**BL2**	**Total**
Date survey (2017)	Start	May 2	Sept 11	May 4	Sept 14	
	End	May 12	Nov 22	June 1	Nov 21	
Sampled individuals		130	132	130	131	
Age range (years)		18–67	19–66	19–65	19–65	
Median age		45.5	47.0	44.0	44.0	
Mean age ± 95% CI		43.8 ± 2.1	45.1 ± 2.1	43.9 ± 1.9	44.8 ± 1.8	
Females (F)		39	34	12	16	
Males (M)		91	98	118	115	
Paired samples		PD, *n* = 69		BL, *n* = 97		
Travel abroad in the	Country with	19	30	14	29	96
preceding 6-months	*Ae. albopictus*					
	Country with no	7	4	7	6	20
	*Ae. albopictus*					
	Not specified	1	4	–	–	5
	No travel	103	94	109	96	402
	Total	130	132	130	131	523
Cutaneous reaction (0–5)	Low (0–1)	73	77	61	92	303
	Mid (2–3)	49	49	18	25	141
	High (4–5)	8	3	4	3	18
	Total	130	129	83	120	462
Number of bites (0-5)	Low (0–1)	117	53	124	64	358
	Mid (2–3)	13	49	6	59	127
	High (4–5)	0	28	0	8	36
	Total	130	130	130	131	521
Timing of bites	Day	11	61	12	38	122
	Night	7	22	7	43	79
	Day and night	6	46	7	36	95
	Total	24	129	26	117	296

### Entomological Monitoring

**Figure 1:**
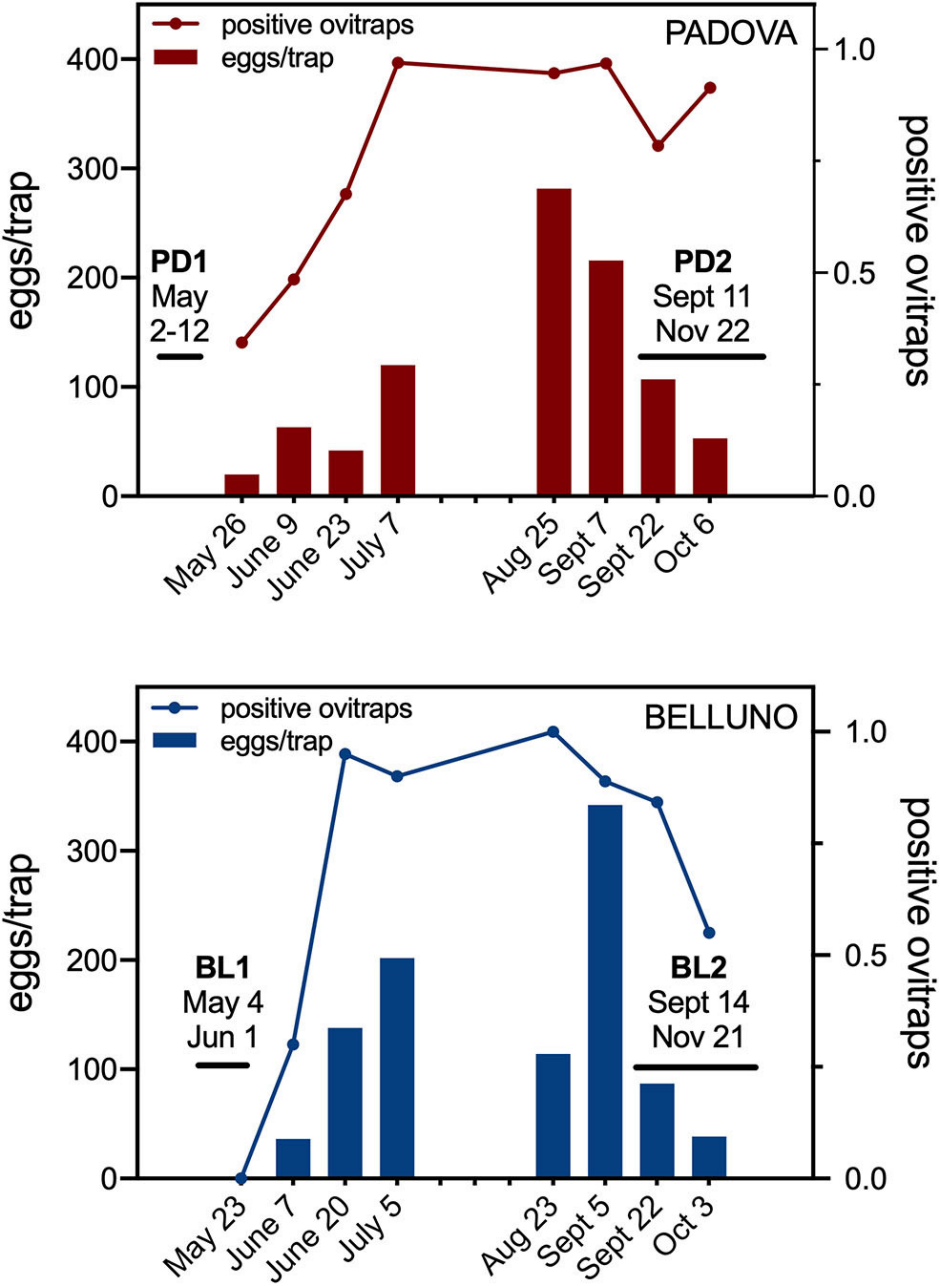
Entomological monitoring by ovitraps in the study areas. Bars show the average number of eggs per positive ovitraps (eggs/trap, left Y axis). Lines represent the proportion of positive ovitraps, i.e., the number of ovitraps with eggs over the total number of ovitraps (positive ovitraps, right Y axis). The time intervals for the two sera collections in each study area are reported.

Oviposition traps were placed in Padova and Belluno (Supplementary Figures 1B,C) in the time frame between the two sera collections in order to provide an estimation on the relative population dynamics of *Ae. albopictus*. Both the mean number of eggs per ovitrap and the percentage of positive ovitraps indicated that mosquitoes started appearing around the last week of May in Padova (19.9 eggs/ovitrap, 34.4% positive ovitraps) and shortly later, around the first week of June, in Belluno (36.3 eggs/ovitrap, 30.0% positive ovitraps). The number of eggs per ovitrap progressively increased during the summer period reaching a peak the last week of August in Padova (281.4 eggs/ovitrap, 94.6% positive ovitraps) and first week of September in Belluno (342.1 eggs/ovitrap, 88.9% positive ovitraps) and decreasing afterwards (Figure 1). Despite the original assumption of Padova being an area of higher *Ae. albopictus* density than Belluno, ovitraps data did not show a clear difference between the two study sites. On the contrary, the temporal dynamic fully supports the expectations that (i) individuals whose sera were collected before summer were not significantly exposed to *Ae. albopictus* bites for at least 4–5 months and (ii) individuals surveyed after summer were naturally exposed to the tiger mosquito during the warm months, from June to September.

### IgG Responses to *Ae. albopictus* Salivary Gland Extracts

**Figure 2:**
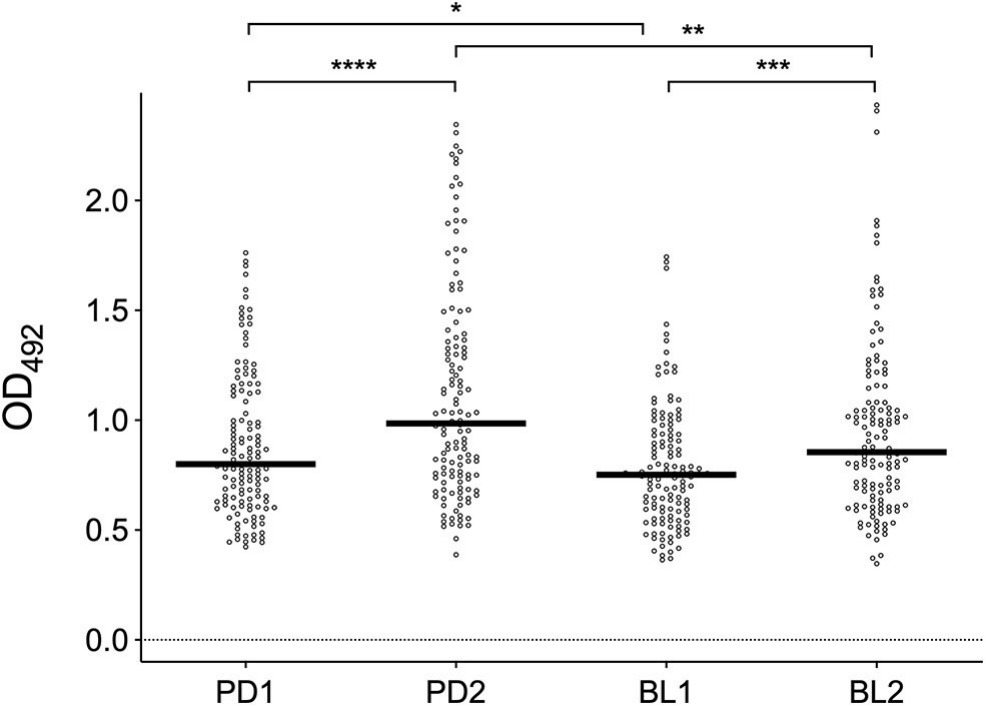
IgG responses to *Ae. albopictus* salivary gland protein extracts. Anti-SGE IgG levels in participants to the four different surveys (PD1, PD2, BL1, and BL2) as indicated at the bottom. IgG levels are expressed as OD values. Number of individuals for each survey according to Table 1. Dots mark the individual values and horizontal bars represent the medians. Significant difference in the pairwise comparisons (Mann-Whitney U test) is also reported: *p < 0.05; **p < 0.01; ***p < 0.001; ****p < 0.0001.

IgG antibody responses against mosquito saliva or salivary gland extracts have been previously shown to reliably reflect the intensity of human exposure to bites of either *Anopheles* or *Aedes* species (Remoue et al., 2006; Orlandi-Pradines et al., 2007; Fontaine et al., 2011; Doucoure et al., 2012b, 2014). Therefore, we first analyzed the IgG responses to *Ae. albopictus* SGE in sera collected before and after summer in the two study areas. Anti-SGE IgG responses were significantly higher in sera collected after the summer period of high mosquito density both in Padova (PD2) and Belluno (BL2) as compared to those collected before summer in the same areas (Padova, *p* < 0.0001; Belluno, *p* = 0.0009; Figure 2). Moreover, IgG antibody levels against SGE were higher in Padova than Belluno during both before (*p* = 0.0341) and after (*p* = 0.0070) the high-density mosquito seasons. These observations, in contrast to ovitraps data, seems to confirm the original assumption of Padova being an area of higher exposure to *Ae. albopictus* than Belluno.

### IgG Responses to *Ae. albopictus* 34k2 Salivary Protein

**Figure 3:**
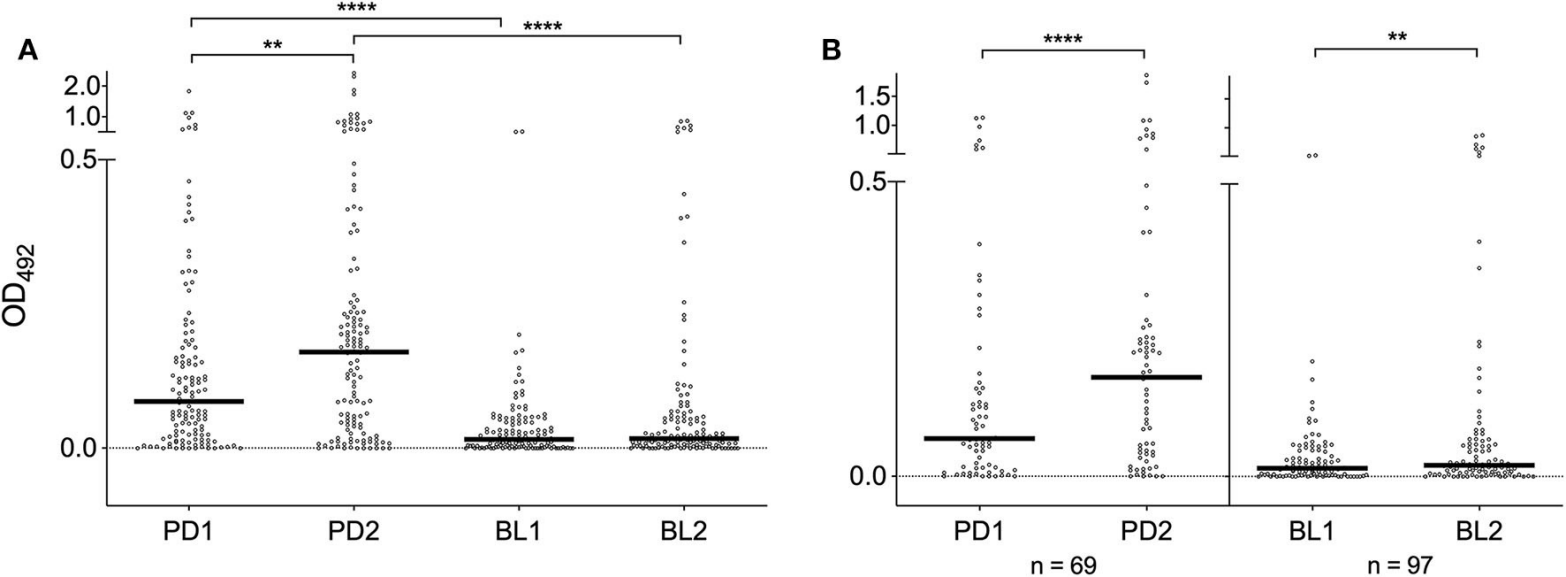
IgG responses to the *Ae. albopictus* salivary gland protein al34k2. **(A)** Anti-al34k2 IgG levels in all participants to the four different surveys. IgG levels, number of individuals, dots, bars and p values as in Figure 2. **(B)** IgG responses in paired samples from Padova (left panel) and Belluno (right panel). The number of individuals is indicated at the bottom. Dots and bars as in **(A)**. Significant difference in the pairwise comparisons (Wilcoxon matched-pairs test): **p < 0.01; ****p < 0.0001.

IgG responses to SGE and al34k2 showed a weak, but clearly positive correlation (Spearman *r* = 0.43, 95% CI 0.36–0.50, *n* = 523, *p* < 0.0001). When the different surveys were compared, a seasonal variation of IgG levels was found in Padova (*p* = 0.0043) and anti-al34k2 IgG responses were higher in Padova than in Belluno both before (*p* < 0.0001) and shortly after the summer season (*p* < 0.0001). Comparison of anti-al34k2 IgG antibody levels between the two sets of sera collected in Belluno failed to show a significant seasonal variation (Figure 3A). However, when only paired samples from the two localities were analyzed (i.e., those individuals whose sera were collected both in the first and the second survey), a significant seasonal increase was found not only in Padova (*n* = 69, *p* < 0.0001) but also in Belluno (*n* = 97, *p* = 0.0032) (Figure 3B). Overall, despite the relatively weak correlation, anti-al34k2 IgG responses exhibited a pattern of variation fully consistent with the anti-SGE IgG responses. Therefore, these observations convincingly suggest that IgG responses to al34k2 may be suitable to assess spatial and temporal variation of human exposure to bites of the tiger mosquito *Ae. albopictus*.

### IgG1 and IgG4 Responses to *Ae. albopictus* 34k2 Salivary Protein

**Figure 4:**
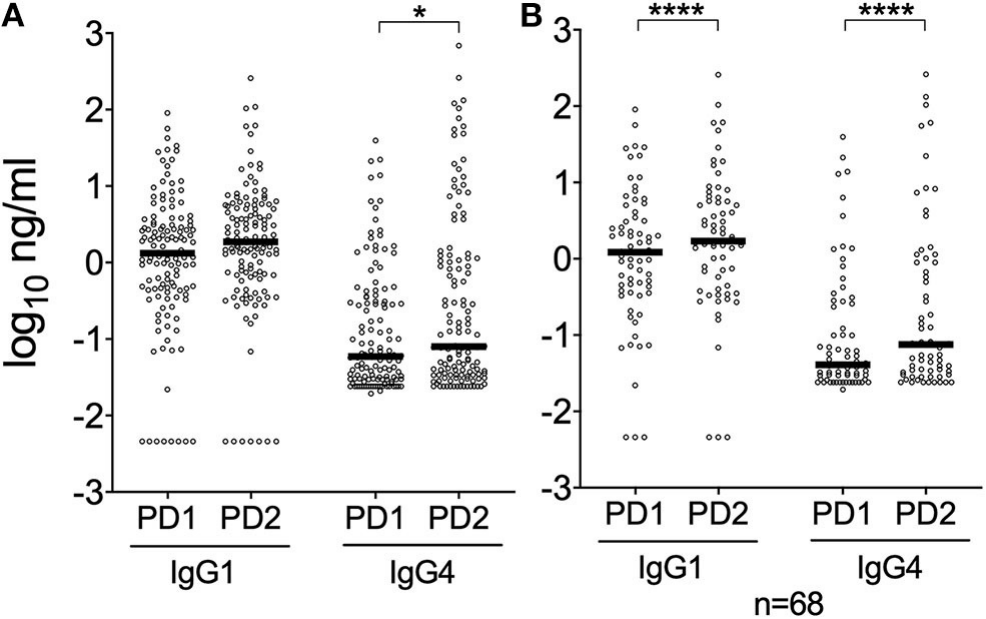
IgG1 and IgG4 responses to the *Ae. albopictus* salivary gland protein al34k2 in Padova. **(A)** Individual anti-al34k2 IgG1 and IgG4 levels in participants to the PD1 (*n* = 128) and PD2 (*n* = 128) surveys. IgG1 and IgG4 levels are expressed in ng/ml. Dots, bars and *p*-values as in Figure 2. **(B)** IgG1 and IgG4 responses against al34k2 in paired samples from Padova (*n* = 68). IgG1 and IgG4 levels, dots and bars as above. Pairwise comparisons by the Wilcoxon matched-pairs test (*p < 0.05; ****p < 0.0001).

Previous studies showed that the *An. gambiae* gSG6 and cE5 salivary proteins induce in naturally exposed individuals differential antibody responses, with the gSG6 antigen evoking high levels of IgG4 antibodies and cE5, on the contrary, triggering an IgG1-dominated response (Rizzo et al., 2014a,b). To get insights into IgG subclass-specificity of antibody responses to the al34k2 protein we determined IgG1 and IgG4 antibody titers in the sera collected in Padova before (PD1) and after (PD2) the high density mosquito season. As expected a positive correlation was found between anti-al34k2 IgG and IgG1 levels (Spearman *r* = 0.64, 95% CI 0.56–0.70, *n* = 262 *p* < 0.0001), and similar results were obtained for IgG and IgG4 (Spearman *r* = 0.68, 95% CI 0.61–0.74, *n* = 262, *p* < 0.0001) levels. Median IgG1 titers appeared to be over ten-fold higher than corresponding IgG4 titers in both surveys (Figure 4A; *p* < 0.0001). A highly significant increase of both anti-al34k2 IgG1 and IgG4 levels was observed in PD2 by pairwise comparisons between paired samples (*n* = 68, *p* < 0.0001; Figure 4B); instead, only IgG4 levels showed a weakly significant increase after summer when all samples were considered (*p* = 0.0326, Figure 4A). These results clearly indicate that antibody responses against the al34k2 salivary protein show a large predominance of IgG1 antibodies.

### IgG Responses to *Ae. albopictus* SGE and al34k2 According to Age

Individual IgG responses to *Ae. albopictus* SGE and to al34k2 were also analyzed according to age. Overall, a negative correlation was found between age and IgG responses to *Ae. albopictus* SGE or al34k2. Spearman's rank correlation analysis indicated a clear trend of antibody responses to decrease with age for both Padova surveys, especially when considering the anti-SGE IgG responses; on the contrary, in Belluno a weakly significant negative correlation was only found for the anti-al34k2 IgG responses in the BL2 survey (Table 2, Supplementary Figure 3A). This general trend was confirmed when participants to the surveys were divided in four different age groups (18–30, 31–40, 41–50 and >50 years old). Pairwise comparisons indicated a clear and significant decrease with age of the anti-SGE and anti-al34k2 IgG responses in Padova; again, this was not the case for Belluno where some decrease was only observed in the over 50 years old category in the BL2 survey (Supplementary Figure 3B).

**Table 2:** Correlation between age and IgG levels.

	**Spearman r**	**95% CI**	***n***	***p*-value**
PD1-SGE	−0.3861	−0.53 to −0.22	130	<0.0001
PD2-SGE	−0.4570	−0.59 to −0.31	132	<0.0001
BL1-SGE	0.0007	−0.18 to 0.18	130	ns
BL2-SGE	−0.1648	−0.33 to 0.01	131	ns
PD1-al34k2	−0.2548	−0.42 to −0.09	130	0.0030
PD2- al34k2	−0.2435	−0.40 to −0.07	132	0.0049
BL1- al34k2	−0.0961	−0.27 to 0.08	130	ns
BL2- al34k2	−0.2149	−0.38 to −0.04	131	0.0137

To make sure that age distribution did not represent a source of bias we compared the age of the different cohorts of individuals (PD1, PD2, PD paired, BL1, BL2, BL paired) by the Kruskal-Wallis or Mann-Whitney tests and found no significant difference. We also calculated the frequencies of the four age groups in the different cohorts (Supplementary Figure 4A) and compared them by the Chi-square test without finding any difference (chi-square 16.37, df 15, *p* = 0.358). Finally, we also compared IgG levels before and after the mosquito season in the two sites by age groups, using both paired and unpaired samples. Median anti-SGE and anti-al34k2 IgG levels were higher after the summer season in almost all the pairwise comparisons (30/32), even though statistical significance was only reached in ~40% of cases (56% for SGE and 25% for al34k2, Supplementary Figure 4B), likely because of the relatively small sample size. Overall, these observations suggest that age should be taken into consideration in similar studies but also indicate that it does not appear to be a relevant source of bias in our investigation.

### Anti-saliva IgG Responses and Individual Perception of Exposure to Mosquito Bites

Participants to the surveys, along with the informed consent, were asked to fill a short questionnaire on their individual perception of cutaneous reaction to mosquito bites, intensity/number of bites and timing of occurrence (Table 1). Despite the intrinsic limitations of this subjective self-assessment, we verified the possible correlation with anti-SGE and anti-al34k2 IgG antibody levels. Overall, individuals reporting mid to high (2–5) cutaneous reactions showed higher anti-SGE IgG levels as compared to those with absent or low (0–1) reactions. This was supported by the observation that median OD values, as well as 25th and 75th percentiles, were in most cases (PD1, PD2, and BL2) higher for the mid-to-high category, although the difference reached statistical significance only for the PD1 survey (*p* = 0.0036; Supplementary Figure 5A). IgG responses to both *Ae. albopictus* SGE and to al34k2 were also compared in individuals reporting a low number of bites (score 0–1) versus those accounting for mid to high number of bites (score 2–5). For both antigens, the 25th and 75th percentiles and median OD values were, in the very large majority of cases, higher in the mid to high category; however, statistical significance was only found when considering the IgG response to SGE in the PD1 (*p* = 0.0075) and BL1 surveys (*p* = 0.0320, Supplementary Figure 5B). No general common trend and/or significant difference was recognizable when IgG responses were compared in individuals reporting mainly day- versus night-time bites, only exception being the BL2 survey where anti-al34k2 IgG levels were slightly higher (*p* = 0.0280) in individuals accusing a larger number of bites during daytime.

## Discussion

In the last decades the tiger mosquito *Ae. albopictus* impressively expanded its geographic distribution colonizing areas with relative cool climates and gaining the reputation of one of the most invasive species worldwide (Bonizzoni et al., 2013; Lwande et al., 2019). Further spreading in both United States and Europe has been predicted for the next decades (Kraemer et al., 2019), with a consequent expansion of the population at risk from mosquito-borne diseases as dengue, yellow fever, chikungunya and Zika. In this context the cases of autochthonous transmission in Europe (Gossner et al., 2018; Brady and Hay, 2019) and the large outbreaks in the Reunion Island (Renault et al., 2007; Vincent et al., 2019) sound as an alarm bell calling public health authorities for improved monitoring and control of the tiger mosquito.

Historically, the control of vector populations and the reduction of human-vector contact have been the most important weapons in the fight against mosquito-borne diseases. More recently, the identification of genus-specific mosquito salivary proteins (Ribeiro et al., 2010; Arcà and Ribeiro, 2018) paved the way for the development of serological toolboxes that may allow for the simultaneous evaluation of human-vector contact and circulation of pathogens transmitted by the same vector. Such a tool may be useful for epidemiological studies and the evaluation of both transmission risk and efficacy of vector control interventions. The successful outcome of studies with *Anopheles* malaria vectors stimulated efforts to develop similar markers for *Aedes* vectors, leading to the identification of the Nterm-34kDa peptide as candidate biomarker of human exposure to *Ae. aegypti*, with some encouraging indications also for *Ae. albopictus* (reviewed by Sagna et al., 2018).

We previously obtained promising evidence of the immunogenicity of the al34k2 salivary protein to both mice and humans (Buezo Montero et al., 2019). With the aim to validate this antigen as candidate marker of exposure to *Ae. albopictus*, we measured the anti-al34k2 IgG responses in sera collected from adult healthy individuals, before and after the summer high-density mosquito season, in two different areas colonized by the tiger mosquito. It is worth pointing out that, apart from the Nterm-34kda peptide, the availability of additional markers of exposure to the tiger mosquito may prove useful for several reasons. First, the Nterm-34kDa peptide is designed on the *Ae. aegypti* 34k1 salivary protein and the corresponding peptide from the *Ae. albopictus* ortholog is rather divergent (12/19 identical residues with a 3 amino acids gap). This may imply a relatively low sensitivity, which might be a limiting factor in conditions of low mosquito density, when also traditional entomological approaches become less reliable. Second, human immune responses to mosquito salivary antigens exhibit significant individual variability, as shown for the *An gambiae* gSG6 and cE5 (Rizzo et al., 2014a). As a consequence, multiple antigens may be very helpful providing a more comprehensive view and eventually increasing the sensitivity and/or specificity of the immunoassays.

When we measured the IgG responses to *Ae. albopictus* SGE we found, as expected, a significant increase from low to high mosquito density period. A similar pattern was found for the anti-al34k2 IgG responses: in both study sites they increased shortly after the summer exposure and declined after the winter period of non-exposure to *Ae. albopictus*, even though this was more evident in Padova than in Belluno (Figure 3). This is an important finding in view of two considerations. First, an effective marker should evoke an IgG response sufficiently short-term to detect variation in human-vector contact, for example from high to low vector density season or after the implementation of vector control measures. Second, the duration of IgG responses is salivary antigen-dependent, as previously reported for the *An. gambiae* gSG6 and cE5 (Rizzo et al., 2014a). The correlation between individual responses to SGE and al34k2 was positive, as expected, but relatively weak (Spearman *r* = 0.43). However, taking into account (i) that saliva is a complex mixture of more than hundred salivary proteins, (ii) the lower sensitivity of anti-al34k2 IgG responses, and (iii) the individual variability in the response, this observation does not seem really surprising and should not be negatively interpreted. Overall, the observations reported here suggest that in natural conditions the anti-al34k2 IgG responses appear suitable to evaluate seasonal variations of human exposure to the tiger mosquito and eventually to assess the efficacy of vector control interventions at reducing the host-vector contacts.

To our surprise, entomological monitoring by ovitraps did not show the expected difference between the two study sites. Nevertheless, anti-SGE and anti-al34k2 IgG responses were higher in Padova than Belluno both before and after summer, a result that is perfectly in line with the original assumption. Providing a simple and unequivocal explanation to this discrepancy is not easy; however, a few considerations should be kept in mind. First, the correlation between number of eggs and adult females density is not so straightforward and may be affected by several different variables (Manica et al., 2017). Second, the presence of *Ae. koreicus*, whose eggs are practically indistinguishable from those of *Ae. albopictus* (Montarsi et al., 2015), may have contributed to the high numbers found in Belluno. Third, ovitraps were placed within the cities (Supplementary Figure 1), but transfusional centers accept blood donors from the whole province. Around 25% of Belluno blood donors were resident in small villages where the tiger mosquito is either absent or present at very low density (Montarsi et al., 2015); on the contrary, all the villages in Padova province are well known to be infested since decades (Romi et al., 1999). In addition, IgG responses to mosquito saliva have been previously shown, in different settings, to be a reliable indicator of host exposure to mosquito bites (Remoue et al., 2006; Orlandi-Pradines et al., 2007; Fontaine et al., 2011; Doucoure et al., 2012a, 2014) and, differently from entomological measures, provide a direct indication of human-vector contact. For these reasons, overall, we feel rather confident suggesting that IgG responses to the al34k2 salivary protein appear a reliable marker also to detect spatial variation of human exposure to *Ae. albopictus*.

Human IgG responses to mosquito saliva are mainly characterized by antibodies of the IgG1 and IgG4 subclasses and very low IgG2 and IgG3 concentrations. High levels of antigen-specific IgG4 antibodies may be related to allergenic properties of insect salivary proteins (Peng and Simons, 2004); they may also be associated with immune tolerance, as is the case for beekeepers who carry elevated levels of venom-specific IgG4 antibodies with anti-inflammatory potential (Garcia-Robaina et al., 1997; Varga et al., 2013). Moreover, higher levels of IgG4 antibodies against *Ae. aegypti* saliva were recently reported in dengue-infected individuals in an endemic area in Colombia (Cardenas et al., 2019). We determined anti-al34k2 IgG1 and IgG4 levels in individuals from the PD1 and PD2 surveys and, in both cases, median IgG1 titers were at least 10-fold higher than corresponding IgG4 levels (Figure 4). A similar finding was previously reported for the *An. gambiae* salivary protein cE5 in naturally exposed individuals from a malaria hyperendemic area of Burkina Faso; however, the same individuals carried high levels of anti-gSG6 IgG4 antibodies. This differential responses to the cE5 and gSG6 proteins has been interpreted as a possible indicator of Th1-type and Th2-type polarized immune responses, respectively (Bretscher, 2014; Rizzo et al., 2014a,b). Our findings suggest that the *Ae. albopictus* al34k2 protein may be of limited allergenicity and induces in naturally exposed individuals an IgG1-dominated antibody response that may be indicative of a Th1-type polarization.

IgG antibody responses to SGE and al34k2 showed a trend to decrease with age. This was pronounced in sera collected in Padova but only barely detectable in those from Belluno (Table 2, Supplementary Figure 3). Human cutaneous reactions and antibody responses to mosquito saliva are known to change over time with the continued natural exposure, most likely because of immune tolerance and progressive desensitization to mosquito salivary proteins (Mellanby, 1946; Feingold et al., 1968; Peng and Simons, 1998, 2004; Doucoure et al., 2012b; Cardenas et al., 2019; Montiel et al., 2020). However, while IgG responses to mosquito saliva (a cocktail of ~100–150 proteins) decrease with age, the situation with individual salivary proteins is antigen-dependent. For example, a decrease with age was previously reported for the *An. gambiae* gSG6 (Poinsignon et al., 2009; Rizzo et al., 2011b, 2014b; Montiel et al., 2020) and for *Anopheles albimanus* salivary peptides (Londono-Renteria et al., 2020); on the contrary, a positive correlation was found for the *An*. gambiae cE5 (Rizzo et al., 2014a) and the *Ae. aegypti* D7s4 (Ribeiro et al., 2007; Londono-Renteria et al., 2018). Moreover, intensity and persistence of exposure plays a role in natural desensitization to salivary antigens (Mellanby, 1946; Feingold et al., 1968), and this may explain the different trends we observed in Padova, an area colonized since more than 25 years, and in Belluno, where individuals were exposed to *Ae. albopictus* bites for no more than 5 years.

There are still some possible improvements and a few interesting open questions that wait for answers. First, the absence of unexposed controls prevented the determination of a threshold for seropositivity, which could provide very useful information on seroprevalence. Considering the widespread distribution of the tiger mosquito, getting reliable non-exposed controls may be not so straightforward. Nevertheless, their inclusion in future studies would be very important, especially in view of the encouraging results reported here. Second, it will be of interest to clarify the species-specificity of anti-al34k2 IgG responses. The 34k2 salivary proteins from *Ae. aegypti* and *Ae. albopictus* are 62% identical. However, sera of mice experimentally immunized to *Ae. aegypti* saliva do not carry IgG antibodies recognizing the al34k2 protein. Vice versa, sera from mice exposed to *Ae. albopictus* do not recognize the *Ae. aegypti* orthologous protein ae34k2, which suggests absence of cross-reactivity in the mouse model (Buezo Montero et al., 2019). It will be very interesting to measure the anti-al34k2 IgG responses in naturally exposed individuals living in areas where *Ae. aegypti* is abundant but *Ae. albopictus* is absent. Third, it will be of great value to verify the suitability of the al34k2 antigen for the evaluation of control interventions against the tiger mosquito as for example accomplished by Elanga Ndille and collaborators at La Reunion Island (Elanga Ndille et al., 2016). Finally, it would be very intriguing the validation of the al34k2 antigen in epidemiological settings where arboviral transmission is endemic and maintained by *Ae. albopictus*.

In conclusion, we believe that our study provides promising indications that IgG antibody responses to the *Ae. albopictus* 34k2 salivary protein may be exploited for the evaluation of human exposure to the tiger mosquito. It remains to be established if anti-al34k2 IgG responses represent a species-specific marker or may be useful as more general indicator of exposure to *Aedes* vectors. Serological assays as the one described here have the advantage of providing a direct measure of human-vector contact, not only at the community but also at the individual level, and may be useful to assess the efficacy of vector control interventions in interrupting this interaction. Such a complementary tool can also be employed for epidemiological studies and possibly for estimation of transmission risk. This may be especially helpful when implementation of classical entomological methods is difficult (low vector density, logistic constraints, limited resources, etc.) or when the simultaneous determination of exposure to vector and to specific circulating pathogen(s) by serological measurements may be needed.

## Data Availability Statement

The datasets generated for this study are available on request to the corresponding author.

## Ethics Statement

This study involved healthy human volunteers and the protocol was reviewed and approved by the Ethics Committee of Sapienza University (306/17 RIF.CE: 4479, April 10th 2017). The volunteers provided their written informed consent to participate in this study.

## Author Contributions

BA conceived the study. FM, MP, GC, and BA designed the experiments. SB and FM performed the experiments. PG and FF provided the al34k2 antigen. AlB, SC, GD, and AnB provided the sera. SB, FM, MP, GC, and BA analyzed the data. SB, GC, and BA performed the statistical analysis. BA wrote the first draft of the manuscript. All authors contributed to manuscript revision, read and approved the final submitted version of the manuscript.

## Conflict of Interest

The authors declare that the research was conducted in the absence of any commercial or financial relationships that could be construed as a potential conflict of interest.
