# Supplementary material for: IgG Antibody Responses to the Aedes albopictus 34k2 Salivary Protein as Novel Candidate Marker of Human Exposure to the Tiger Mosquito
Source: Front Cell Infect Microbiol. 2020 Jul 29;10:377. doi: 10.3389/fcimb.2020.00377 (PMC7405501; doi:10.3389/fcimb.2020.00377)
Supplement: Supplementary file 1 [file Data_Sheet_1.PDF]

Supplementary Material

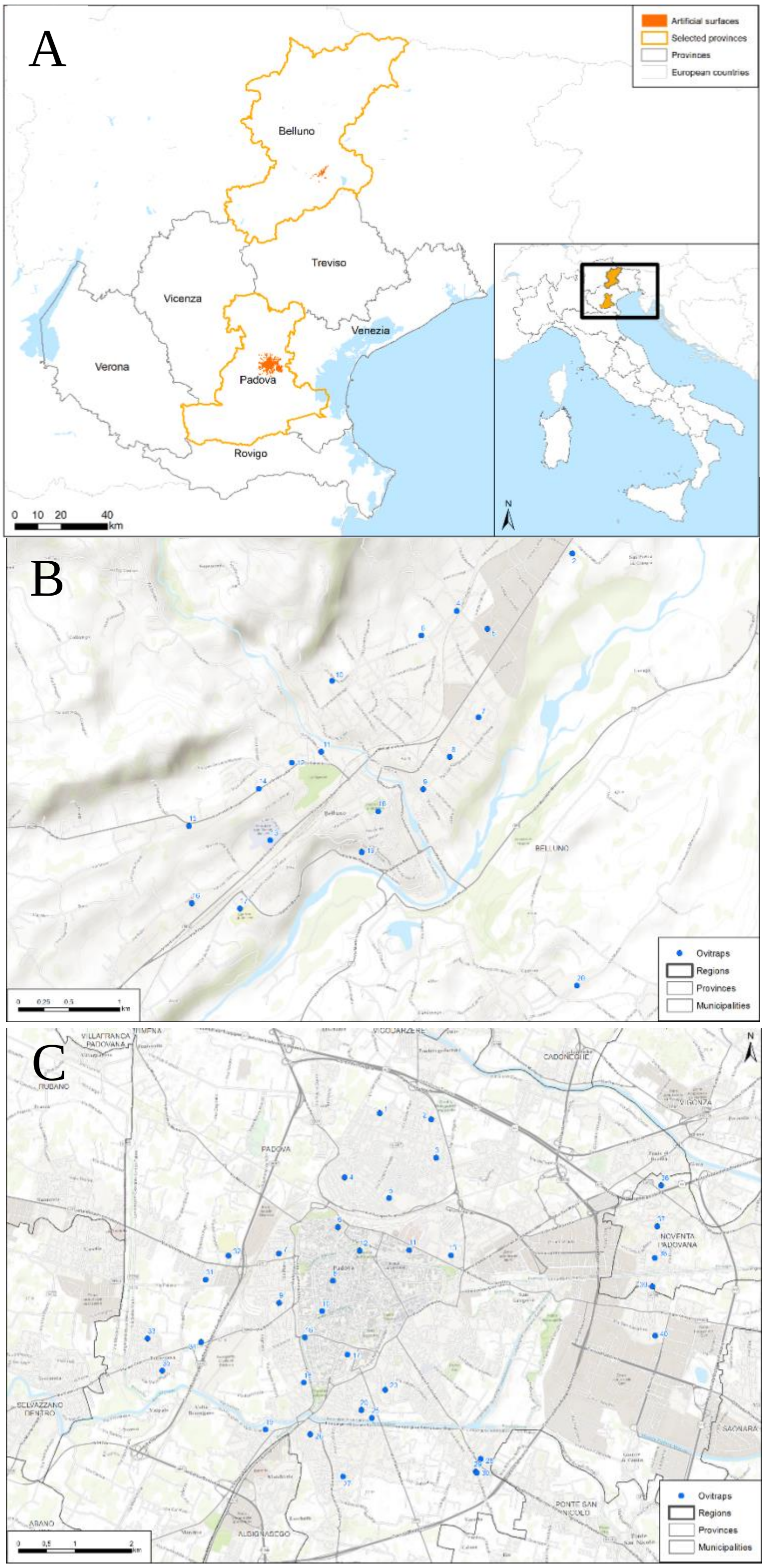

Supplementary Figure 1. Map of the study sites and ovitraps position. (a) Map of the Veneto region: the boundaries of the Belluno and Padova provinces (yellow) and the cities of Padova and Belluno (orange spots) are shown. The inset shows the location of Veneto in Northeast Italy and the Belluno and Padova provinces (yellow areas). **(b)** Map of Belluno and ovitraps position (blue dots). **(c)** Map of Padova and ovitraps position (blue dots).

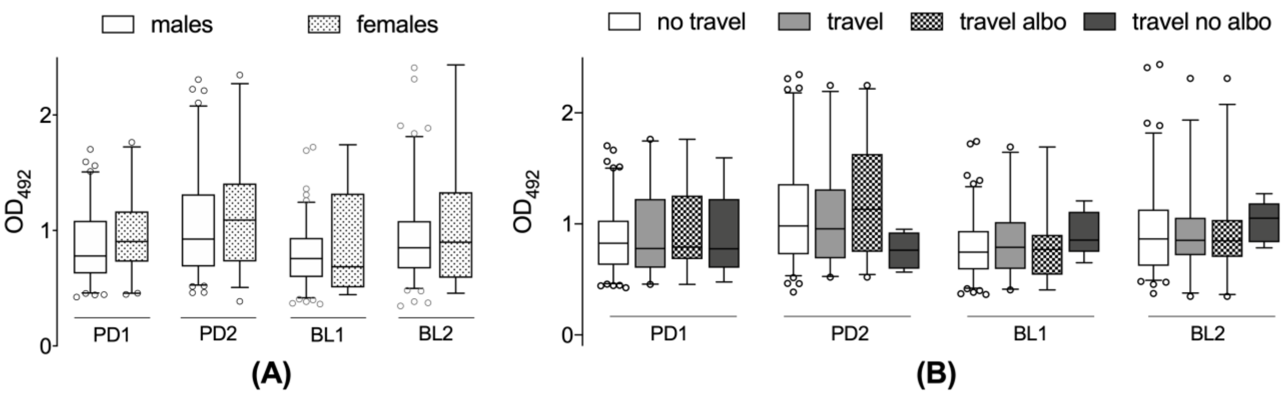

Supplementary Figure 2. IgG responses to *Ae. albopictus* SGE according to sex and travelling. (**A**) Anti-SGE IgG levels in males and females in the four different surveys. (**B**) Anti-SGE IgG levels in individuals who, in the six months before the survey, did not travel abroad (no travel), travelled abroad (travel), travelled to countries where *Ae. albopictus* was present (travel albo) or absent (travel no albo). IgG levels are expressed as OD values. The four different surveys (PD1, PD2, BL1 and BL2) are indicated at the bottom. Boxes display median OD values, 25th and 75th percentiles; whiskers represent 5th and 95th percentiles and dots the outliers. Number of individuals for each survey according to Table1. In all cases pairwise comparisons within each survey showed no significant difference (Mann-Whitney U test, p value >0.05).

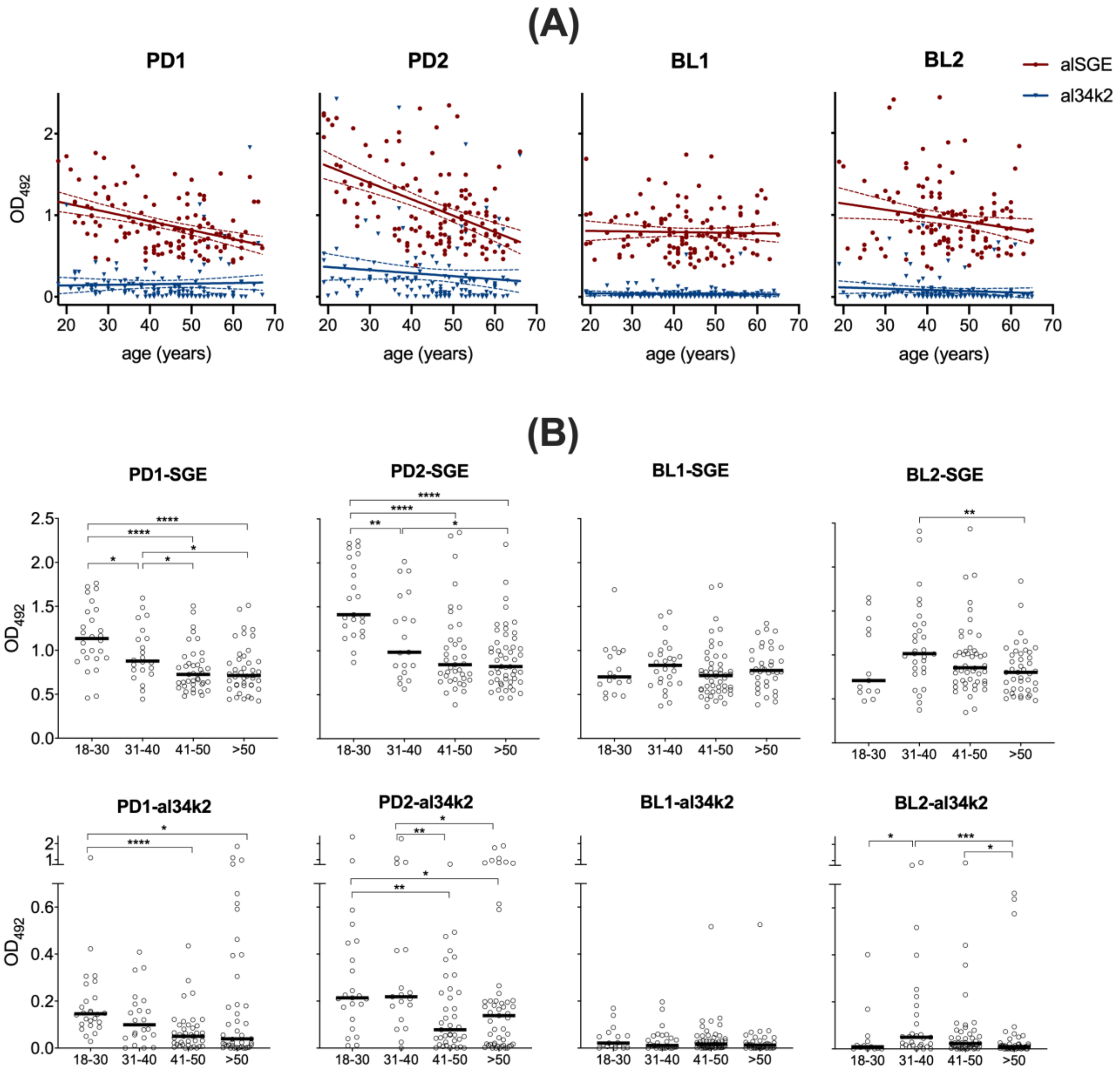

Supplementary Figure 3. Anti-SGE and anti-al34k2 IgG antibody responses according to age. **(A)** The scatter plots show the IgG responses to SGE (red) and al34k2 (blue) as function of age in participants to the four different surveys. Best-fit lines (solid lines) and confidence interval bands (dashed lines) are shown. Results of correlation analysis are reported in Table 2. **(B)** Anti-SGE and anti-al34k2 IgG responses in the four age-groups and different surveys are shown in the upper and lower panel, respectively. IgG levels are expressed as OD values. Dots mark the individual values and horizontal bars represent the medians. Number of individuals in the different age groups as follows: PD1 (18-30, n=26; 31-40, n=22; 41-50, n=39; >50, n=43), PD2 (18-30, n=23; 31-40, n=19; 41-50, n=39; >50, n=51), BL1 (18-30, n=17; 31-40, n=27; 41-50, n=50; >50, n=36), BL2 (18-30, n=13; 31-40, n=29; 41-50, n=47; >50, n=42). Significant difference in the pairwise comparisons (Mann-Whitney U test) is reported: *, p<0.05; **, p<0.01; ***, p<0.001; ****, p<0.0001.

(A)

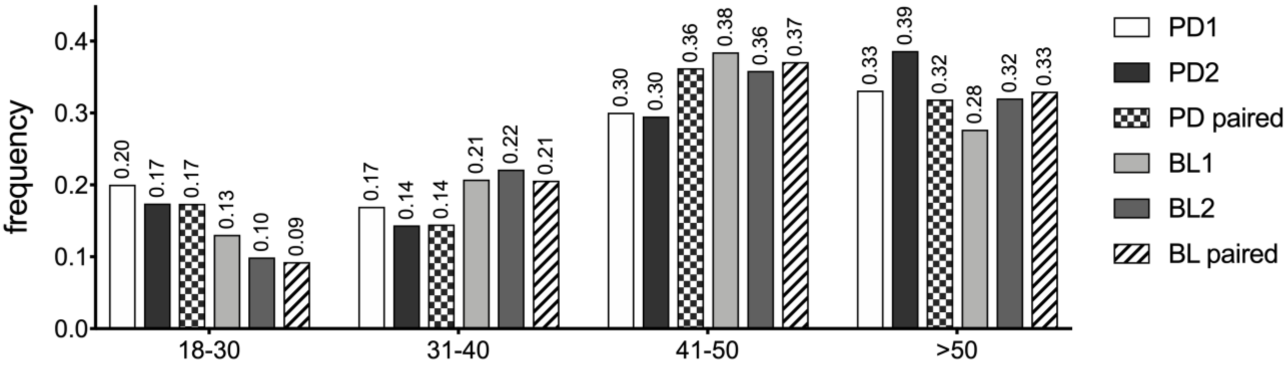

(B)

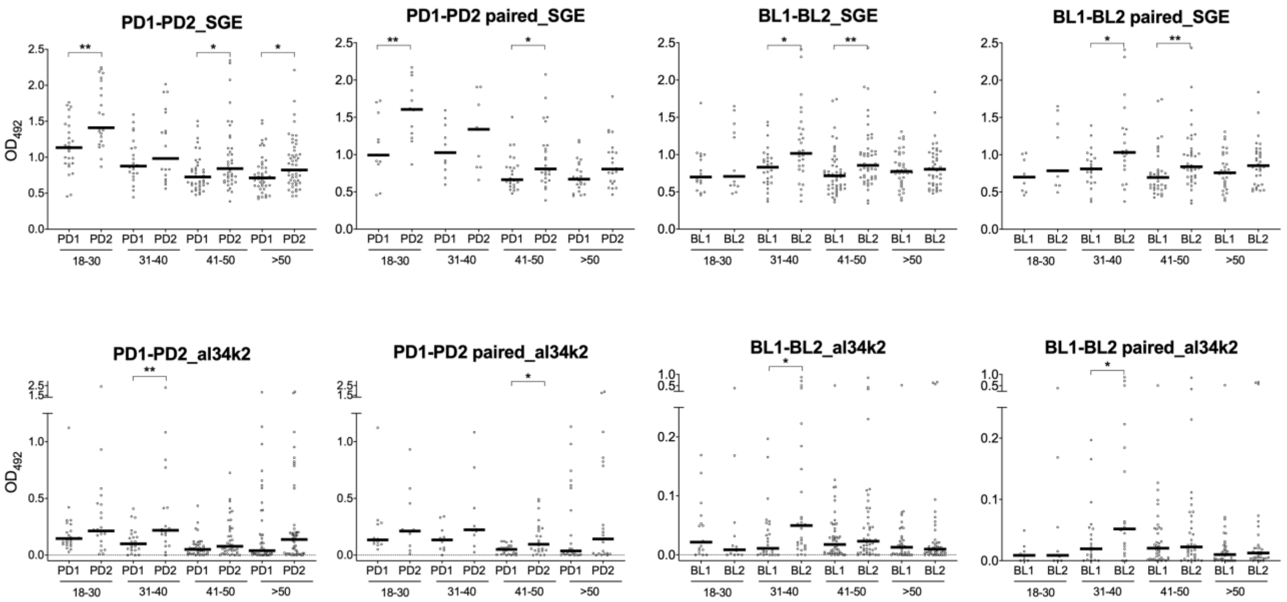

Supplementary Figure 4. Distribution of participants to the different surveys by age groups and comparison of the IgG antibody responses before and after the summer season according to age. (A) Proportion of individuals belonging to the four age groups (18-30, 31-40, 41-50, >50 years old) in unpaired (PD1, PD2, BL1 and BL2) and paired (PD paired, BL paired) samples. **(B)** Comparison of anti-SGE and anti-al34k2 IgG responses before and after the high density mosquito season in the four age groups and different surveys as indicated. IgG levels are expressed as OD values. Dots mark the individual values and horizontal bars represent the medians. Number of individuals per age group in the unpaired samples as in Supplementary Figure 3. Number of individuals per age group in the paired samples as follows: PD paired (18-30, n=12; 31-40, n=10; 41-50, n=25; >50, n=22), BL paired (18-30, n=9; 31-40, n=20; 41-50, n=36; >50, n=32). Significant difference in the pairwise comparisons (Mann-Whitney U test) is reported: *, p<0.05; **, p<0.01.

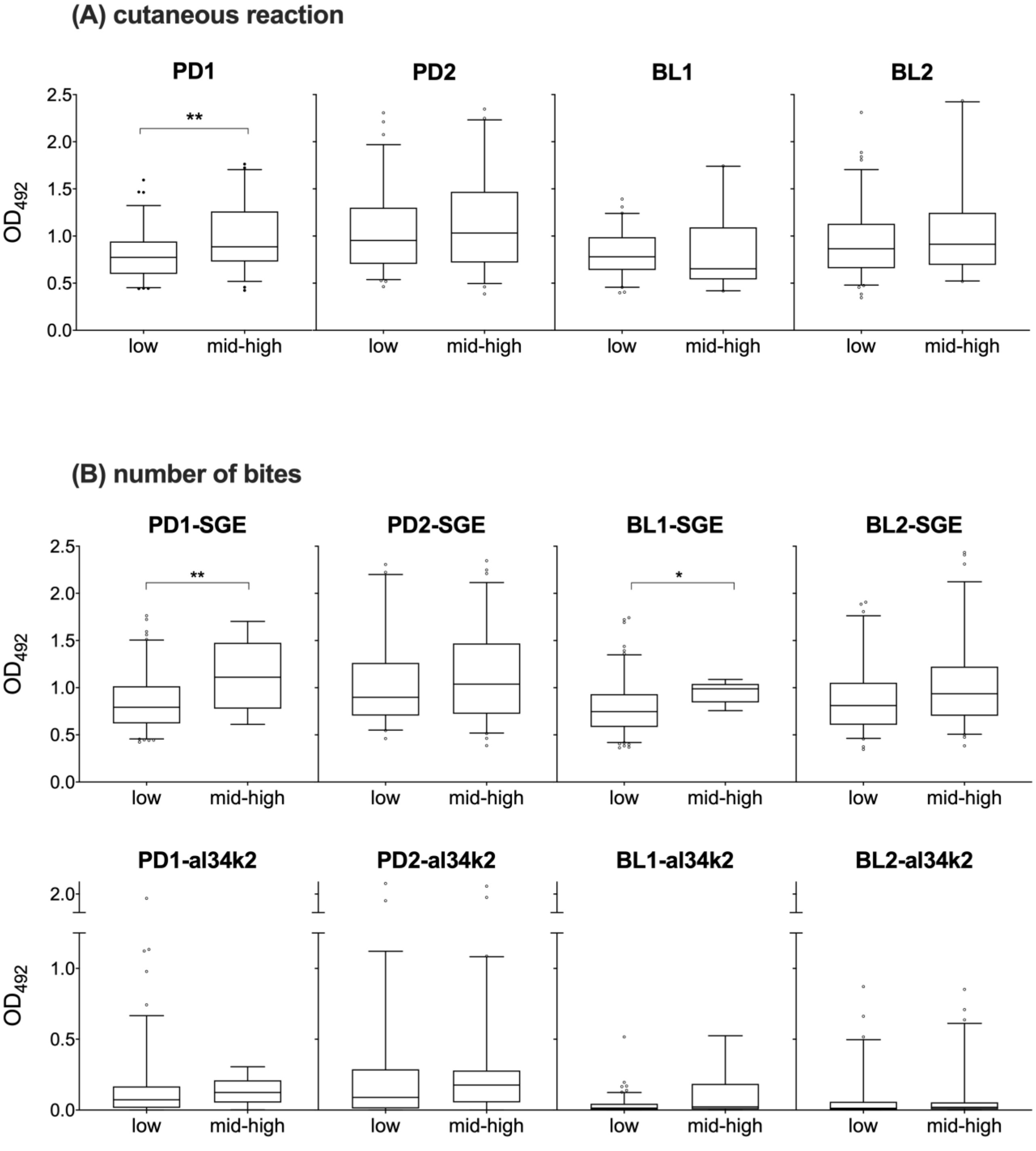

Supplementary Figure 5. IgG responses to salivary antigens and individual perception of mosquito bites. (**A**) IgG responses to *Ae. albopictus* SGE according to intensity of cutaneous reactions to mosquito bites. Boxplots of OD values among individuals in the PD1, PD2, BL1 and BL2 surveys. Boxes display median OD values, 25th and 75th percentiles; whiskers represent 5th and 95th percentiles, dots the outliers. Low=0-1, absent to low intensity reaction (PD1, n=73; PD2, n=77; BL1, n=61; BL2, n=92). Mid-high=2-5, moderate to intense reaction (PD1, n=57; PD2, n=52; BL1, n=22; BL2, n=28). Pairwise comparisons by Mann-Whitney U test. (**B**) IgG responses to *Ae. albopictus* SGE and to al34k2 in the four surveys according to the subjective perception of intensity of mosquito bites: low=0-1, low number of bites, (PD1, n=117; PD2, n=53; BL1, n=124; BL2, n=64); mid-high=2-5, moderate to very high number of bites, (PD1, n=13; PD2, n=77; BL1, n=6; BL2, n=67). Boxplots and pairwise comparisons as above.
